# Bladder Exposure to *Gardnerella* Activates Host Pathways Necessary for *Escherichia coli* Recurrent UTI

**DOI:** 10.3389/fcimb.2021.788229

**Published:** 2021-12-06

**Authors:** Valerie P. O’Brien, Amanda L. Lewis, Nicole M. Gilbert

**Affiliations:** ^1^ Human Biology Division, Fred Hutchinson Cancer Research Center, Seattle, WA, United States; ^2^ Department of Obstetrics, Gynecology and Reproductive Sciences, University of California San Diego, San Diego, CA, United States; ^3^ Department of Pediatrics, Division of Infectious Diseases, Washington University in St. Louis School of Medicine, St. Louis, MO, United States

**Keywords:** urinary tract infection (UTI), bacterial vaginosis (BV), Nur77, immediate early gene expression, bladder, urinary microbiome, orphan nuclear receptor 4A1 (NR4A1), RNA-seq - RNA sequencing

## Abstract

Recurrent urinary tract infections (rUTI) are a costly clinical problem affecting millions of women worldwide each year. The majority of rUTI cases are caused by uropathogenic *Escherichia coli* (UPEC). Data from humans and mouse models indicate that some instances of rUTI are caused by UPEC emerging from latent reservoirs in the bladder. Women with vaginal dysbiosis, typically characterized by high levels of *Gardnerella* and other anaerobes, are at increased risk of UTI. Multiple studies have detected *Gardnerella* in urine collected by transurethral catheterization (to limit vaginal contamination), suggesting that some women experience routine urinary tract exposures. We recently reported that inoculation of *Gardnerella* into the bladder triggers rUTI from UPEC bladder reservoirs in a mouse model. Here we performed whole bladder RNA-seq to identify host pathways involved in *Gardnerella-*induced rUTI. We identified a variety host pathways differentially expressed in whole bladders following *Gardnerella* exposure, such as pathways involved in inflammation/immunity and epithelial turnover. At the gene level, we identified upregulation of Immediate Early (IE) genes, which are induced in various cell types shortly following stimuli like infection and inflammation. One such upregulated IE gene was the orphan nuclear receptor *Nur77* (aka *Nr4a1*). Pilot experiments in *Nur77^-/-^* mice suggest that Nur77 is necessary for *Gardnerella* exposure to trigger rUTI from UPEC reservoirs. These findings demonstrate that bladder gene expression can be impacted by short-lived exposures to urogenital bacteria and warrant future examination of responses in distinct cell types, such as with single cell transcriptomic technologies. The biological validation studies in *Nur77^-/-^* mice lay the groundwork for future studies investigating Nur77 and the Immediate Early response in rUTI.

## Introduction

In humans, the urinary tract is the second most common site of infection, most frequently by uropathogenic *Eschericia coli* (UPEC) (Foxman, 2014). Approximately 1% of bladder infections (cystitis) progress to more serious kidney infections (pyelonephritis), and some of these become life-threatening systemic infections (Ki et al., 2004; Jolley et al., 2012). Recurrent urinary tract infections (rUTIs) are very common: 24% of women with an initial UTI will have rUTI within 6 months, and up to 70% will have rUTI within 1 year (Foxman et al., 2000; Foxman, 2014). Approximately 1% of all women (~35 million worldwide) have six or more rUTIs each year (Foxman et al., 2000; Foxman et al., 2002; Foxman, 2014). These women are often given prophylactic antibiotics, contributing to the problem of antibiotic resistance.

It is now appreciated that UPEC has an intracellular niche within the bladder. Multiple studies of UTI and rUTI in adults and children have identified intracellular UPEC, both in bladder epithelial cells shed in urine and in bladder biopsies (Elliott et al., 1985; Rosen et al., 2007; Robino et al., 2014; Liu et al., 2016; De Nisco et al., 2019). One study using confocal microscopy detected intracellular bacteria in 36.8% of samples from children with acute UTI. Notably, a medical chart review demonstrated that the children with intracellular bacteria were significantly more likely to have a history of rUTI (OR, 8.0; 95% CI, 2.3-27.4) (Robino et al., 2014). In C57BL/6 mice, UPEC can persist within bladder epithelial cells for months after inoculation, without bacteriuria (bacteria in urine) and despite antibiotic treatment (Mulvey et al., 2001; Schilling et al., 2002; Eto et al., 2006; Mysorekar and Hultgren, 2006). In patients, most rUTIs (as many as 82%) are caused by a UPEC strain identical to that of the previous infection (Ejrnaes et al., 2006; Czaja et al., 2009; Luo et al., 2012; Skjot-Rasmussen et al., 2013; Koljalg et al., 2014), even when appropriate antibiotic therapy is given. This could reflect reintroduction of UPEC to the bladder from the gut or vaginal reservoir, but is also consistent with the concept that rUTI can be caused by emergence of the initially infecting UPEC strain from an intracellular bladder reservoir (Mulvey et al., 2001; Hickling and Nitti, 2013; Glover et al., 2014). The potential for bladder reservoirs to seed rUTI has prompted preclinical investigations into strategies to eliminate bladder reservoirs as a means of rUTI. *Gardnerella* strain JCP8151B causes epithelial exfoliation in the vagina in a mouse model (Gilbert et al., 2013). We hypothesized that *Gardnerella* could likewise cause bladder epithelial (urothelial) exfoliation and thus be a trigger of UPEC rUTI from bladder reservoirs in women. This hypothesis was further supported by three clinical observations potentially linking *Gardnerella* with UTI. First, women with bacterial vaginosis, a vaginal dysbiosis in which the vagina is overpopulated by a polymicrobial mixture of bacteria including *Gardnerella*, have an up to 13-fold higher likelihood of UTI than those with *Lactobacillus*-dominated vaginal microbiotas (OR 2.21-13.75) (Harmanli et al., 2000; Hillebrand et al., 2002; Sharami et al., 2007; Sumati and Saritha, 2009; Amatya et al., 2013). Second, women with rUTI who received vaginal interventions that influence the vaginal microbiota (e.g., vaginal estrogen, probiotic intravaginal *Lactobacillus crispatus*) experienced fewer rUTIs than those who received placebo (Stapleton et al., 2011; Rahn et al., 2014; Sadahira et al., 2021). Third, multiple urinary microbiome studies have detected *Gardnerella* in urine collected directly from the bladder (*via* suprapubic aspiration or catheterization), suggesting that *Gardnerella* routinely gains access to the bladder in some women (Wolfe et al., 2012; Pearce et al., 2014; Pearce et al., 2015; Thomas-White et al., 2016; Gottschick et al., 2017). Intriguingly, *Gardnerella* can co-aggregate with UPEC in an *in vitro* biofilm assay (Castro et al., 2016), suggesting possible synergy between these bacterial species.

To test our hypothesis, we developed a mouse model to determine whether *Gardnerella* could cause exfoliation in the bladder and UPEC emergence from bladder reservoirs (Gilbert et al., 2017; O’Brien et al., 2020). We found that in mice with intracellular UPEC reservoirs in the bladder, two transurethral *Gardnerella* inoculations triggered UPEC emergence from reservoirs, leading to rUTI. *Gardnerella* exposure caused urothelial apoptosis and exfoliation, which likely enabled UPEC to emerge from reservoirs. Notably, *Gardnerella* did not stably colonize the bladder, demonstrating that a brief exposure was sufficient to elicit UTI pathogenesis. Here we sought to further characterize the effect of *Gardnerella* bladder exposures in order to identify host processes involved in UPEC rUTI that could serve as potential biomarkers or therapeutic targets. We performed RNA sequencing (RNA-seq) on bladders harboring intracellular UPEC reservoirs, with and without exposure to *Gardnerella*. We found that host gene expression changes following *Gardnerella* exposure were modest, in keeping with a bacterial exposure that does not result in stable colonization. However, gene set enrichment analyses revealed upregulation of many host pathways. Some of the pathways, such as apoptosis and inflammatory cytokines, corroborated the phenotypic results we previously reported in this model, and other pathways were generally related to UTI. Five genes in the Immediate Early response pathway were significantly upregulated, including *Nur77* (aka *Nr4a1*). Notably, Nur77^-/-^ mice were protected from *Gardnerella*-induced rUTI, confirming its role in this model.

## Results

### Mouse Model of Gardnerella Bladder Exposure

**Figure 1:**
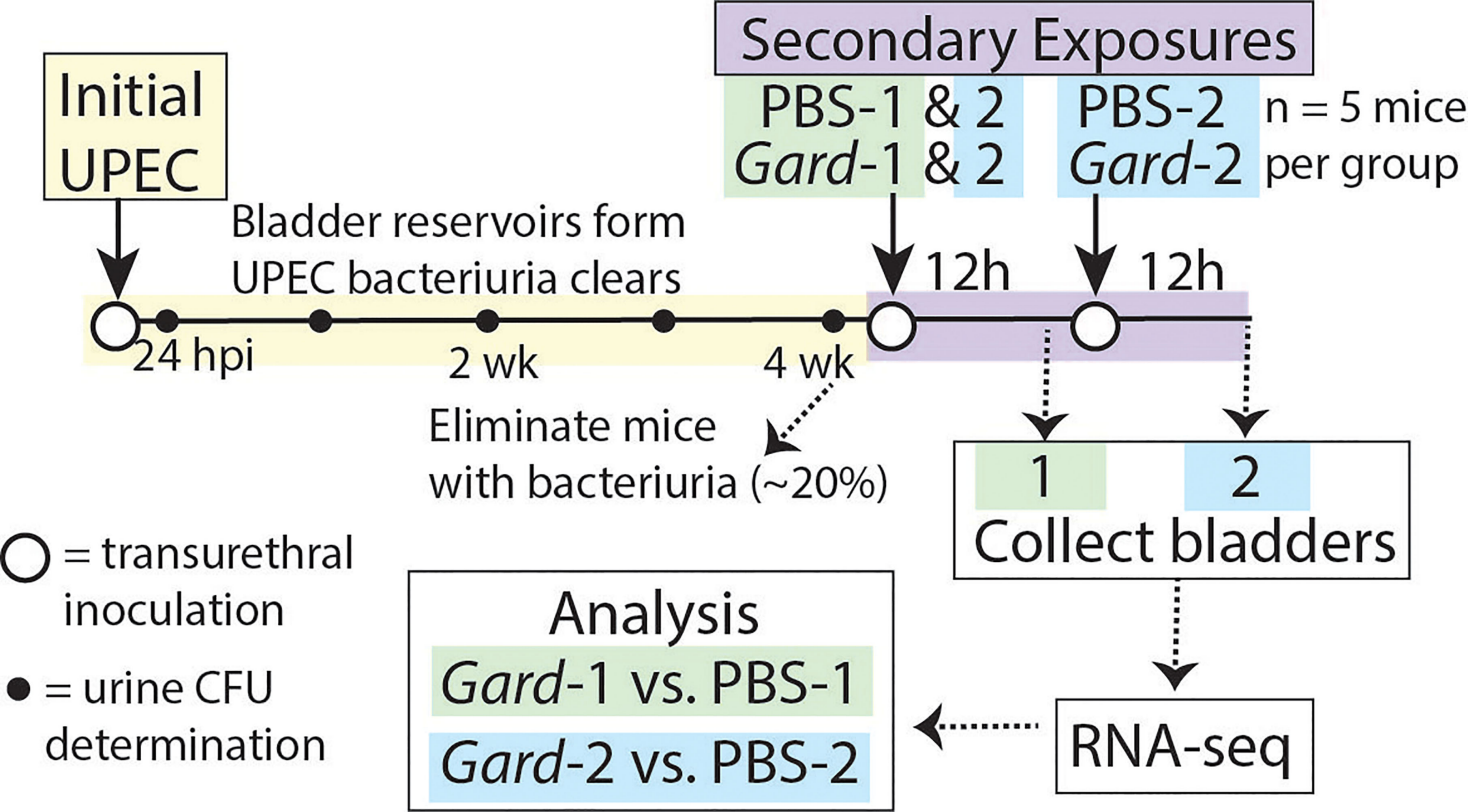
Mouse model schematic for RNA-seq experiments. The time line of the model depicts the ‘Initial UPEC’ reservoir formation phase in yellow and the ‘Secondary Exposure’ phase in purple. Female C57BL/6 mice were inoculated transurethrally (open circles) with UPEC and bacteriuria was monitored weekly (closed circles). Mice that cleared UPEC bacteriuria were inoculated transurethrally with either PBS as a control or *Gardnerella*. Twelve hours later Exposure Group 1 was sacrificed while Exposure Group 2 received an additional transurethral inoculation and was sacrificed twelve hours (h) later. For the bladder titer experiments described in **Figures 4**, **5** the second exposure occurred one week later instead of 12 h later and bladders were collected 72 h after the second exposure.

We performed RNA-seq on whole bladders to identify early host responses to *Gardnerella* bladder exposure that could contribute to UPEC rUTI. **Figure 1** outlines the experimental timeline. First, we established UPEC bladder reservoirs by administering an initial intravesical inoculation (directly into the bladder through the urethra) of 10^7^ colony-forming units (CFU) of the UPEC strain UTI89. Mice that cleared UPEC bacteriuria by 4 weeks post infection (wpi), as determined by weekly urine dilution plating and CFU enumeration, were inoculated intravesically with *Gardnerella* strain JCP8151B or vehicle (PBS) as a control. Our previous experiments demonstrated that *Gardnerella* is cleared by 12 hours (h), so we refer to this as an ‘exposure’ rather than an infection. One group of mice received a single exposure and bladders were collected 12 h later in order to examine early host responses to *Gardnerella*. These groups are labelled PBS-1 and *Gard*-1. Our previous studies demonstrated that two exposures to *Gardnerella* were required to cause urothelial exfoliation and to trigger UPEC rUTI. Therefore, to understand the host responses to *Gardnerella* that could ultimately lead to exfoliation and rUTI, another group of mice, labelled PBS-2 and *Gard*-2, received two exposures, 12 h apart, and bladders were collected 12 h after the second exposure (Gilbert et al., 2017; O’Brien et al., 2020). Consistent with our previous report that UPEC emergence from reservoirs usually occurs 24-72 h after *Gardnerella* exposure, only one mouse in the current study (in the *Gard-*1 group) had detectable reservoir emergence indicated by UPEC titers in urine collected immediately prior to sacrifice.

RNA was extracted from bladders from each experimental group (5 mice per group) and used for RNA-seq to assess host gene expression changes as a result of *Gardnerella* exposures. A total of 640,624,040 RNA-seq reads were generated. Of these, 457,166,961 could be aligned to the *Mus musculus* reference genome. Further details of the RNA-seq reads are found in **Supplementary Table 1**. Principal component analysis and multi-dimensional scatter plots did not reveal obvious clustering of any of the individual exposure groups (not shown).

### Gene Set Enrichment Analysis Shows Pathways Related to Urothelial Turnover and Inflammation

**Figure 2:**
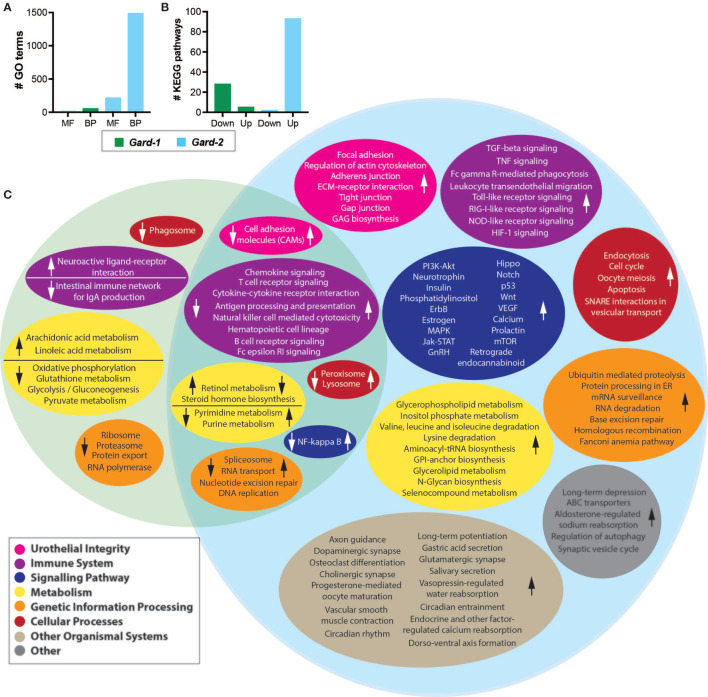
Differential expression analysis. Shown are the host gene expression pathways that were differentially expressed following each *Gardnerella* (Gard) exposure. **(A)** The indicates number of Gene Ontology (GO) terms were significantly up- or down-regulated. MF, molecular functions; BP, biological processes. **(B)** The indicated number of KEGG pathways were significantly up- or down-regulated following *Gardnerella* exposure. **(C)** The Venn diagram displays KEGG pathways that were significantly different between PBS-1 and *Gard*-1 (left, green circle) or PBS-2 and *Gard*-2 (right, blue circle). Pathways with similar functions are organized into color-coded ovals as indicated in the figure legend. The direction of the change in the *Gard* group relative to the PBS group is indicated by arrows in each colored oval. The overlapping section of the Venn diagram indicates pathways impacted in both comparisons, with the arrows on the left in each colored oval indicating the direction of the change for *Gard*-1 and the arrow on the right indicating the direction of the change for *Gard-*2.

We determined which gene ontology (GO) terms were differentially expressed following each *Gardnerella* exposure relative to PBS controls from the same time points (i.e. PBS-1 *vs. Gard*-1 and PBS-2 *vs. Gard*-2). Four GO molecular functions and 54 GO biological processes were significantly down-regulated (FDR adjusted P < 0.05, log_2_FC > 2) following the first *Gardnerella* exposure (**Figure 2A**). The majority of the affected pathways were related to host immune and inflammatory processes (**Supplementary Tables 2, 3**). More substantial differences were seen following the second *Gardnerella* exposure (**Figure 2A**): 215 GO molecular functions and 1,488 GO biological processes were significantly up-regulated (FDR adjusted P < 0.05, log_2_FC > 2). In addition to immune and inflammatory processes, many GO functions were related to urothelial integrity and turnover (**Supplementary Tables 4, 5**). Also, 33 GO terms related to apoptosis were increased, which is consistent with our previous observation of increased cleaved Casp-3 staining and TUNEL-positive urothelial cells following two *Gardnerella* exposures (Gilbert et al., 2017).

### KEGG Pathway Analysis Shows a Dynamic Response to Gardnerella Exposure

We performed a similar gene set analysis (comparing PBS-1 *vs. Gard*-1 and PBS-2 *vs. Gard*-2) using the KEGG Pathway database. Echoing the results from the GO term analysis, more substantial changes occurred following two *Gardnerella* exposures. Compared to one *Gardnerella* exposure, two exposures resulted in a greater number of pathways affected and greater with greater fold changes (**Figures 2B, C** and **Table 1**). Our previous studies showed that two exposures are necessary to elicit significant urothelial exfoliation and rUTI, which is consistent with greater changes to gene expression in the bladder after the second *Gardnerella* exposure.

**Table 1:** Host Pathways Most Affected by *Gardnerella* Bladder Exposures.

	KEGG Pathway	logFC	P-value (uncorr)
***Gard-1 vs.* PBS-1**	mmu00830 Retinol metabolism	2.6486	4.42E-03
	mmu00591 Linoleic acid metabolism	2.4315	8.55E-03
	mmu03010 Ribosome	-8.4850	3.91E-15
	mmu00190 Oxidative phosphorylation	-4.7135	2.03E-06
	mmu04060 Cytokine-cytokine receptor interaction	-3.9074	5.39E-05
	mmu04062 Chemokine signaling pathway	-3.8554	7.19E-05
	mmu04612 Antigen processing and presentation	-3.7393	1.36E-04
	mmu03050 Proteasome	-3.0583	1.60E-03
	mmu04145 Phagosome	-2.8300	2.48E-03
	mmu04672 Intestinal immune network for IgA production	-2.8545	2.82E-03
	mmu04660 T cell receptor signaling pathway	-2.7785	3.10E-03
	mmu04650 Natural killer cell mediated cytotoxicity	-2.6919	3.87E-03
	mmu03040 Spliceosome	-2.5963	5.14E-03
	mmu04514 Cell adhesion molecules (CAMs)	-2.5036	6.43E-03
	mmu00480 Glutathione metabolism	-2.3857	1.00E-02
***Gard-2 vs.* PBS-2**	mmu04510 Focal adhesion	6.2557	6.93E-10
	mmu04144 Endocytosis	6.1367	1.25E-09
	mmu04120 Ubiquitin mediated proteolysis	5.3357	1.68E-07
	mmu04151 PI3K-Akt signaling pathway	5.0988	2.34E-07
	mmu04722 Neurotrophin signaling pathway	4.8336	1.74E-06
	mmu04810 Regulation of actin cytoskeleton	4.6898	1.95E-06
	mmu04520 Adherens junction	4.8834	2.46E-06
	mmu04141 Protein processing in endoplasmic reticulum	4.6692	2.53E-06
	mmu04910 Insulin signaling pathway	4.6103	3.45E-06
	mmu04070 Phosphatidylinositol signaling system	4.4558	1.16E-05
	mmu04360 Axon guidance	4.2598	1.64E-05
	mmu04012 ErbB signaling pathway	4.2117	2.92E-05
	mmu04110 Cell cycle	4.1265	3.06E-05
	mmu04728 Dopaminergic synapse	4.0484	3.72E-05
	mmu04668 TNF signaling pathway	4.0322	4.84E-05

The pathways that were activated following one *Gardnerella* exposure were primarily related to metabolism, while the pathways with decreased expression included several related to the immune system and signaling (**Supplementary Table 6**). In contrast to one *Gardnerella* exposure, where more pathways were downregulated than upregulated, after two *Gardnerella* exposures all but two of the affected pathways were upregulated (**Supplementary Table 7**). Consistent with our previous observation of urothelial exfoliation at this time point, several affected pathways were related to epithelial integrity and renewal, such as focal adhesion, cell junctions (adherens, tight, gap), actin cytoskeleton regulation, and Wnt signaling. Many of the upregulated pathways were related to mucosal immune responses, as would be expected following a bacterial exposure. In addition to pathways specific for certain immune cell types, such as T cells, NK cells, and B cells, changes were observed for immune processes like phagocytosis, antigen processing and presentation and cytokine/chemokine signaling. Several signaling pathways related to inflammation and immunity were also affected, including PI3K-Akt, NF-κB, Jak-STAT and Hippo.

When considering the temporal dynamics of the pathway changes, we found that 20 pathways were significantly different in expression at both time points, but for each of these the change was in opposite directions (**Figure 2C** arrows; **Table 2**). For example, pathways relating to retinol metabolism and steroid hormone biosynthesis were upregulated after the first exposure but downregulated after the second. In contrast, inflammatory pathways like cytokine-cytokine receptor interaction, chemokine signaling, and antigen processing and presentation were downregulated after the first exposure and upregulated after the second. These results demonstrate that *Gardnerella* elicits a dynamic transcriptional response in the bladder.

**Table 2:** Temporal Dynamics of Pathway Expression Following *Gardnerella* Exposures.

KEGG Pathway	*Gard-*1 *vs.* PBS-1 logFC	*Gard*-2 *vs.* PBS-2 logFC
mmu00830 Retinol metabolism	2.648601	-2.510346
mmu00140 Steroid hormone biosynthesis	1.996097	-1.931811
mmu04060 Cytokine-cytokine receptor interaction	-3.907424	2.897815
mmu04062 Chemokine signaling pathway	-3.855439	3.037386
mmu04612 Antigen processing and presentation	-3.739260	2.294584
mmu04660 T cell receptor signaling pathway	-2.778472	3.455259
mmu04650 Natural killer cell mediated cytotoxicity	-2.691867	3.428895
mmu03040 Spliceosome	-2.596279	3.904566
mmu04514 Cell adhesion molecules (CAMs)	-2.503606	3.139679
mmu00240 Pyrimidine metabolism	-2.328605	2.401758
mmu04640 Hematopoietic cell lineage	-2.206054	1.939485
mmu04064 NF-kappa B signaling pathway	-2.181243	3.326770
mmu04662 B cell receptor signaling pathway	-2.040245	3.059671
mmu03013 RNA transport	-1.933156	3.408601
mmu04664 Fc epsilon RI signaling pathway	-1.852960	2.579883
mmu04142 Lysosome	-1.832163	3.212280
mmu00230 Purine metabolism	-1.815900	2.192536
mmu03030 DNA replication	-1.795758	1.852620
mmu03420 Nucleotide excision repair	-1.774988	2.183002
mmu04146 Peroxisome	-1.750474	2.769018

### Differential Gene Expression Suggests a Role for the Immediate Early Response Pathway

**Figure 3:**
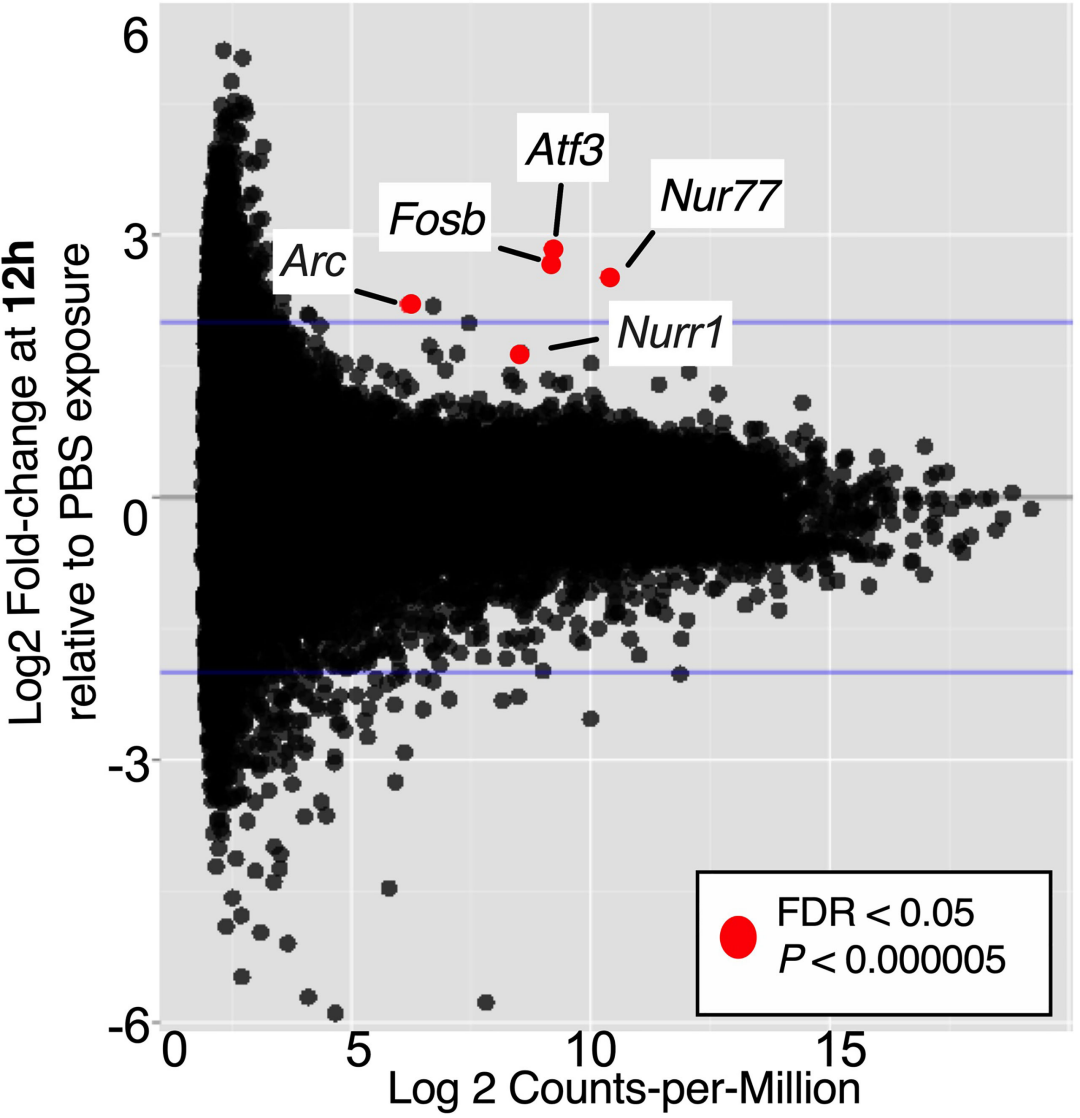
Immediate Early genes upregulated in bladders 12 h after one *Gardnerella* exposure. The volcano plot indicates genes that had significantly higher expression in *Gard*-1 compared to PBS-1 bladders. FDR, false discovery rate correction for multiple comparisons.

It is not atypical for gene set enrichment analyses to uncover more effects than can be detected when assessing significantly differentially expressed individual genes, especially in biologically complex samples like whole organ homogenates. Considering the complexity of the model, which depends on the outcomes of the initial UPEC reservoir formation phase and on the duration of time *Gardnerella* is maintained in the bladder after each inoculation, it was not entirely surprising that few significant differences were detectable when the data were analyzed at the gene level, comparing PBS-1 *vs. Gard*-1 and PBS-2 *vs. Gard*-2. Five genes were significantly increased (FDR adjusted P < 0.05, log_2_FC > 1.6) following the first *Gardnerella* exposure (**Figure 3** and **Supplemental Table 8**), and no genes withstood FDR correction after the second *Gardnerella* exposure. Notably, all of the upregulated genes (*Atf3*, *Fosb*, *Nur77, Nurr1* and *Arc*) belong to the class of Immediate Early (IE) response genes that are rapidly co-induced in multiple cell types in response to external stimuli, such as infection and inflammatory signals (Wilson et al., 2010; Bahrami and Drablos, 2016; Crean and Murphy, 2021). This suggests that the presence of these genes in the dataset was not random or artifactual, but reflects a coordinated rapid response to *Gardnerella* exposure. The fact that these IE genes were increased at the first time point, but not at the second, is consistent with previous reports demonstrating rapid induction and then return to baseline of IE genes (Pei et al., 2005; Tan et al., 2020).

### Nur77 Is Necessary for Gardnerella-Induced UPEC rUTI

**Figure 4:**
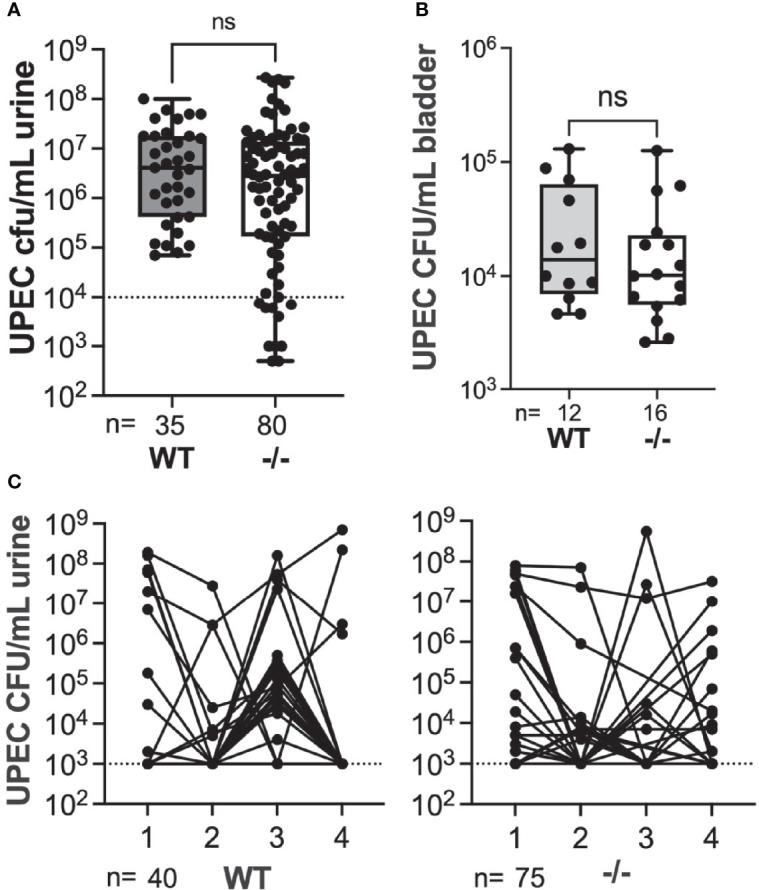
Absence of Nur77 does not affect acute or chronic UPEC bacteriuria or bladder reservoir titers. **(A)** Acute UPEC bacteriuria 24 hpi following initial infection in wild type (WT) mice and mice globally lacking *Nur77* (-/-). **(B)** UPEC titers in bladders collected 2 weeks after initial UPEC infection. All mice had cleared UPEC bacteriuria prior to bladder analysis, thus titers represent intracellular reservoirs. **(C)** UPEC weekly bacteriuria titers following initial UPEC infection. Each dot represents data from an individual mouse. ns, not significant.

The RNA-seq gene-level data suggested that IE genes could play a role in *Gardnerella*-induced UPEC rUTI. The orphan nuclear receptor *Nur77* (also called *Nr4a1*) is expressed early in the IE pathway, acting as a transcription factor for other IE genes including *Atf3* (Yoon et al., 2011; Gao et al., 2016). We used *Nur77* ^-/-^ mice (whole-body) to investigate whether RNA-seq findings could translate to rUTI outcomes. We first established latent UPEC reservoirs in wild type C57BL/6 mice and age-matched *Nur77* ^-/-^ mice on the C57BL/6 background (Lee et al., 1995). Since the role of Nur77 during UTI has not previously been studied, we examined whether the absence of Nur77 impacted UPEC titers during initial infection (yellow bar on **Figure 1**). There was no significant difference in the overall level of acute UPEC bacteriuria at 24 hours post infection (hpi) during the initial infection between the mouse strains (**Figure 4A**), although the proportion of mice with low titers (<10^4^ CFU/mL) was significantly greater in *Nur77* ^-/-^ compared to WT mice (0% WT, 14% *Nur77* ^-/-^; Fisher’s exact P < 0.05). In a subset of animals, we examined UPEC titers during the initial infection phase in bladder tissue following clearance of bacteriuria at 2 wpi (a measure of intracellular reservoirs) and there was no significant difference between WT and *Nur77* ^-/-^ mice (**Figure 4B**). The proportion of mice that had chronic bacteriuria that persisted at > 10^4^ CFU/mL urine at all time points during the initial infection phase out to 4 wpi was indistinguishable between the two groups (2/39 WT, 2/67 *Nur77* ^-/-^) (**Figure 4C**). Together these data demonstrate that Nur77 is not essential for initial UPEC UTI in naïve mice.

**Figure 5:**
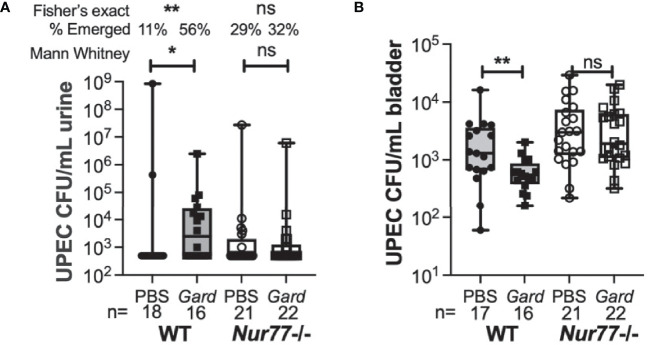
Mice lacking Nur77 are protected from *Gardnerella*-induced UPEC reservoir emergence. Mice harboring bladder UPEC reservoirs were exposed to PBS or *Gardnerella* twice at a one-week interval. Urines were collected daily for three days after the second exposure and mice were euthanized after three days. **(A)** Shown are the highest UPEC urine titers of each individual mouse. **(B)** Bladder UPEC titers are shown. The number of mice in each group is given at the bottom of the graphs. *P < 0.05; **P < 0.01.

Next, we took mice that had cleared initial UPEC bacteriuria by 4 wpi and gave them two secondary exposures, 1 week apart, to *Gardnerella* or PBS. We examined bacteriuria during the subsequent 72 hr after the second exposure as we described previously (Gilbert et al., 2017; O’Brien et al., 2020). We used this exposure model (rather than two exposures 12 h apart, as in the RNA-seq experiment) because it has a higher overall rate of rUTI and because the effects of *Gardnerella* exposure are also evident in the bladder UPEC titers at the experimental endpoint. Similar to our previous studies (Gilbert et al., 2017), the rate of spontaneous emergence in WT PBS control animals was 11% (**Figure 5A**). Although knockout mice appeared to have a somewhat higher baseline of emergence compared to WT, there was no statistically significant difference in rUTI incidence between WT and *Nur77* ^-/-^ mice exposed to PBS (11% WT *versus* 26% *Nur77^-^* ^/-^; P = 0.247, Fisher’s exact test). Consistent with our previous results, *Gardnerella* exposure increased the incidence of UPEC rUTI in WT mice by approximately 5-fold (56% *Gard vs.* 11% PBS in WT mice; P < 0.01). Conversely, the incidence of rUTI was indistinguishable between the *Gardnerella* and PBS exposure groups in *Nur77^-^* ^/-^ mice 32% *Gard vs.* 29% PBS, **Figure 5A**). In line with our previous findings, *Gardnerella-*exposed WT mice had lower bladder UPEC burdens at the experimental endpoint (**Figure 5B**). This is presumably due to egress of UPEC from the tissue concordant with development of bacteriuria. In contrast, in *Nur77* ^-/-^ mice, bladder titers were no different between mice exposed to *Gardnerella* and those exposed to PBS, which is consistent with the urine titer data. Taken together, these results corroborate the RNA-seq data and point to Nur77 as a necessary host factor for *Gardnerella*-induced UPEC rUTI from bladder reservoirs.

## Discussion

Millions of women suffer from recurrent UPEC UTI (Foxman et al., 2000; Foxman, 2014), most commonly caused by the same strain that caused the initial infection (Ejrnaes et al., 2006; Czaja et al., 2009; Skjot-Rasmussen et al., 2013; Koljalg et al., 2014). At least some instances of same-strain rUTI are likely due to UPEC emergence from protected bladder intracellular reservoirs (Mulvey et al., 2001; Hickling and Nitti, 2013; Glover et al., 2014). We and others have demonstrated that UPEC can persist intracellularly in the mouse bladder for weeks or months, and can emerge and elicit rUTI after bladder exfoliation is experimentally induced (Mulvey et al., 2001; Schilling et al., 2002; Eto et al., 2006; Mysorekar and Hultgren, 2006). In our model, we use two *Gardnerella* exposures as the trigger for bladder exfoliation and rUTI (Gilbert et al., 2017; O’Brien et al., 2020). In the present study we demonstrate that *Gardnerella* exposures in mice containing UPEC reservoirs affected the expression of host pathways related to urothelial exfoliation and regeneration, mucosal inflammation and immunity, and other processes. Immediate Early (IE) genes were upregulated following one *Gardnerella* exposure. The orphan nuclear receptor Nur77 is a key IE gene (Yoon et al., 2011; Gao et al., 2016), and we found that whole-body *Nur77* knockout mice were protected from recurrent UPEC UTI following *Gardnerella* exposure – that is to say, the rate of rUTI was not increased in *Nur77* ^-/-^ mice exposed to *Gardnerella* compared to those mice exposed to PBS. Thus, the IE response may contribute to *Gardnerella*-induced recurrent UPEC UTI.

*Gardnerella* has frequently been isolated as the most abundant organism in “urinary microbiome” studies, including those using expanded culture techniques to detect live bacteria (Wolfe et al., 2012; Pearce et al., 2014; Hilt et al., 2014; Pearce et al., 2015; Karstens et al., 2016; Wu et al., 2017). Importantly, these studies collected urine by catheterization or suprapubic needle aspiration to limit vaginal contamination of urine specimens, supporting the conclusion that *Gardnerella* can indeed be found within the bladder. In a recent longitudinal culturomics study of healthy women, *Gardnerella* was often present at high relative abundance in urine sample pairs collected from the same woman at different time points (Ksiezarek et al., 2021). Another large study found hospital inpatients with *G. vaginalis* in their urine were more likely to have a history of rUTI than patients in whom *G. vaginalis* was not detected (Josephson et al., 1988). Although these clinical studies make it clear that *Gardnerella* can be found in the bladder and urine, it is not yet known whether *Gardnerella* stably colonizes the urinary tract, *vs.* being repeatedly re-introduced into the bladder by hygienic and/or sexual behaviors. We have shown that *Gardnerella* does not stably colonize the mouse bladder and is cleared within 12 h of exposure, but nonetheless triggers exfoliation and UPEC rUTI (Gilbert et al., 2017). Here we detected significant changes in expression of several pathways even relatively early after a single *Gardnerella* exposure; more substantial changes occurred after two *Gardnerella* exposures as indicated by significant changes in GO terms and KEGG pathways. These findings further support a model in which repeated introduction of *Gardnerella* into the bladder (which may occur in women after sexual activity) can drive host responses and rUTI phenotypes. Thus, our study provides further support for “covert pathogenesis,” the notion that a brief exposure to a microbe (here, *Gardnerella*) can affect the host enough to drive disease attributed to another microbe (here, *E. coli*) (Gilbert and Lewis, 2019).

Several pathways identified in this RNA-seq analysis are corroborated by phenotypic observations that we previously reported in this mouse exposure model. As would be expected following bacterial exposure, here we observed changes in many host pathways and processes related to the mucosal immune response. The conclusion that *Gardnerella* exposures trigger an inflammatory response in bladders harboring UPEC reservoirs is further supported by our previous finding of higher levels of IL-12, IFN-g, and RANTES in bladder homogenates (Gilbert et al., 2017). Interestingly, pathways related to T and B cells were upregulated after the second *Gardnerella* exposure. This observation could implicate adaptive immunity generated by the initial UPEC infection as a contributor to UPEC reservoir emergence. A previous study showed that an adaptive immune response is necessary to clear a challenge UPEC infection that was introduced by a second UPEC inoculation two weeks after a primary UPEC infection in C57BL/6 mice (Mora-Bau et al., 2015). Other studies have demonstrated that bladder inflammation differs between first and second UPEC exposures and that severe bladder inflammation can impact UTI outcomes (O’Brien et al., 2018; Yu et al., 2019). More work is needed to understand bladder immune responses to *Gardnerella* exposure and UPEC reservoir emergence. The RNA-seq pathway analysis also implicated urothelial integrity and turnover, as well as apoptosis. Our previous studies corroborate these findings, as we have shown that our model strain, *Gardnerella* JCP8151B (also used in the present study), elicits both vaginal (Gilbert et al., 2013; Gilbert et al., 2019) and urothelial exfoliation (Gilbert et al., 2017). In the same urinary tract exposure model used here, mice exposed to *Gardnerella* displayed evidence of apoptosis in the urothelium. Compared to mice exposed to PBS, those exposed to *Gardnerella* had increased urothelial TUNEL staining and cleaved Caspase-3 12 h after two *Gardnerella* exposures (Gilbert et al., 2017). Thus, the RNA-seq findings of pathways related to inflammation and urothelial integrity echo the biological phenotypes we previously reported in the mouse model and the findings from our whole organ analysis support the relevance of RNA-seq methods for identifying biologically relevant host responses to *Gardnerella* bladder exposures.

The model and data presented here have some limitations. Although whole bladder RNA-seq is an established method for investigating host response during UTI (O’Brien et al., 2016; Yu et al., 2019), transcriptional changes cannot be attributed to specific cell types, but rather reflect changes that occurred at the organ level. As well, using the whole organ for bulk RNA-seq may mask gene expression changes that only occur in a small subset of cells, which could be one explanation for the relatively modest number of genes for which expression changes after *Gardnerella* exposure were statistically significant following FDR correction. Nonetheless, the RNA-seq data corroborated previously reported phenotypic findings in this model (apoptosis, inflammatory cytokines) and identified a host gene (*Nur77*) that we subsequently found to be necessary for *Gardnerella*-induced rUTI. These findings support the utility of RNA-seq methods for assessing host responses to even transient bladder microbial exposures and warrant future single-cell transcriptomic studies to specifically examine urothelial, immune, and other bladder cell responses.

The model presented here involves multiple exposures to two different microbes that could independently or synergistically stimulate host responses over time. While this likely reflects the situation occurring in women, it does present some challenges for interpretation. A caveat of the current dataset is that it cannot distinguish changes that required a second *Gardnerella* exposure from changes that resulted from the first exposure, but required additional time to become apparent. However, since we previously reported that a single *Gardnerella* exposure was rapidly cleared and did not cause urothelial exfoliation or UPEC rUTI, it is very likely that most of the pathway upregulation observed here was due to the second exposure, but this should be determined in future studies. Our finding that host gene expression pathways were primarily downregulated after one *Gardnerella* exposure and upregulated after two exposures could suggest that the second time point reflects a host genetic signature of UPEC reservoir reactivation. Additionally, since all of the mice used in the present study harbored UPEC reservoirs (because of our interest in rUTI), we cannot distinguish changes that were due specifically and solely to *Gardnerella* from changes that resulted from “re-awakening” of UPEC reservoirs. Relatively little is understood with respect to how UPEC emerge from quiescent intracellular reservoirs and what host processes are involved. Furthermore, UPEC infection has a lasting effect on the bladder mucosa that is likely to also impact how the bladder responds to subsequent exposures. Consistent with this idea, we previously reported that the bladder cytokine changes caused by *Gardnerella* were distinct between naive mice and those with UPEC reservoirs (Gilbert et al., 2017). Future studies aimed at distinguishing between these various possibilities could examine the bladder transcriptome following *Gardnerella* exposure in naive mice and following induction of UPEC rUTI from reservoirs by other means (e.g. protamine sulfate (Mysorekar and Hultgren, 2006) or chitosan (Blango et al., 2014)).

Our RNA-seq data implicated the nuclear receptor Nur77 (aka Nr4a1) as an early responder to *Gardnerella* exposure. Nur77 regulates myriad cellular processes, that intersect with the UPEC UTI pathogenic cascade and could influence rUTI outcomes. For example, Nur77 regulates apoptosis in multiple tissue types (Rajpal et al., 2003; Herring et al., 2019), and we previously found that *Gardnerella* induced bladder exfoliation *via* apoptosis (Gilbert et al., 2017). Whether or not Nur77 drives exfoliation in the *Gardnerella*-exposed urothelium remains to be determined. Nur77 also modulates inflammation (Rodriguez-Calvo et al., 2017) and has been specifically implicated in T cell responses (Liebmann et al., 2018) and Ly6C- monocytes (Hanna et al., 2011). Nur77 modulated inflammatory responses to *E. coli* in the lung during pneumonia in mice (Cui et al., 2019), but the role of Nur77 in mediating bladder responses to UPEC has not been examined. In the present study we did not conduct an in-depth characterization of acute UPEC UTI in *Nur77* ^-/-^ mice. However, the first phase of our rUTI model entails UPEC infection and monitoring of urine bacterial titers over time during the initial infection phase, prior to secondary exposures. We observed no differences between wild type and *Nur77* ^-/-^ mice in the initial UPEC infection phase of the model. As well, we found that *Nur77* ^-/-^ mice did harbor stable bladder UPEC reservoirs. Thus, *Nur77* may be dispensable for initial UPEC bladder infection in C57BL/6 mice, though we did not assess chronic bladder or kidney infection or other mouse UTI phenotypes. Strikingly, we found that *Nur77* ^-/-^ mice were protected from *Gardnerella*-induced recurrent UPEC UTI, as evidenced by no difference in the incidence of post-exposure bacteriuria or remaining bladder reservoir titers between *Gardnerella*-exposed *vs.* PBS-exposed *Nur77* ^-/-^ mice. In this pilot study, the wild type and *Nur77* ^-/-^ mice were not littermates. Future studies will directly compare *Nur77* ^-/-^ mice to *Nur77* ^+/-^ and wild type littermate controls to validate our rUTI findings. Nur77 is a druggable target, with several ligands being explored for treatment of diseases such as cancer, metabolic disorders, hyperinflammatory responses and endometriosis (Wu and Chen, 2018; Mohankumar et al., 2020). If our findings translate to rUTI in humans, this could open up a therapeutic avenue that is much needed in the current climate of increasing antibiotic resistance (Tamadonfar et al., 2019). Future studies will more closely examine the mechanism(s) for Nur77-mediated response to *Gardnerella* in the bladder, will investigate whether IE responses impact UTI phenotypes in other mouse models, and will test whether Nur77 could also play a role in host response to *Gardnerella* in the vagina.

## Materials and Methods

### Ethics Statement

Mouse experiments were carried out in strict accordance with the recommendations in the Guide for the Care and Use of Laboratory Animals. The Institutional Animal Care and Use Committee (IACUC) of Washington University School of Medicine approved the protocol (Protocol Number: 20170081).

### Bacterial Strains and Growth Conditions

Uropathogenic *E. coli* strain UTI89, harboring a kanamycin resistance cassette (Wright et al., 2005), was grown aerobically at 37°C in static liquid culture in Lysogeny Broth (LB) medium, or on LB agar plates with 25 μg/ml kanamycin. *Gardnerella* strain JCP8151B (Lewis et al., 2013), historically regarded as *G. vaginalis* and recently referred to as *G. piotii* (Hill et al., 2019), was grown anaerobically at 37°C in shaking liquid culture in NYCIII medium, or on NYCIII agar plates with 1 mg/ml streptomycin. Mouse inocula were prepared as previously described (O’Brien et al., 2020).

### Mice

Six- to seven-week-old female C57BL/6 mice (“wild type”) were obtained from Charles River (Fredericks facility). Mice globally deficient in *Nur77* (a.k.a. Nr4a1) were obtained from Jackson Laboratories (B6;129S2-Nr4a1tm1Jmi/J, catalog #006187). Mice were given a regular chow diet in a specific pathogen-free facility with a 12 h light/12 h dark cycle at Washington University School of Medicine. Mice were allowed to acclimate to the facility after transport for 1 week prior to experiments.

### Mouse Urinary Tract Inoculation Experiments for RNA-Seq

Experiments were performed essentially as described previously (Gilbert et al., 2017; O’Brien et al., 2020). Briefly, mice were anesthetized with isoflurane and then inoculated transurethrally with 50 μL prepared 1 x 10^7^ CFU UPEC inoculum. Urine was collected at 24 hpi, and weekly thereafter for 4 weeks. Mice that no longer had detectable UTI89 in urine at 4 weeks, reflecting resolution of the initial bladder lumen infection, were then inoculated transurethrally with 50 uL prepared inoculum of 1 x 10^8^ CFU *Gardnerella* strain JCP8151B (10 mice) or PBS (10 mice). Twelve hours later, five mice from each exposure group (*Gard*-1 and PBS-1) were sacrificed and their bladders were collected aseptically and flash frozen in liquid nitrogen for future RNA isolation. The remaining five mice per group received a second transurethral inoculation with JCP8151B or PBS and were sacrificed another 12 h later to collect bladders for RNA isolation (*Gard*-2 and PBS-2).

### Library Preparation and Sequencing

Bladders were homogenized and RNA was extracted using the RNeasy Plus Mini kit (Qiagen). Libraries were prepared from each bladder individually with 10 ng of total RNA and RNA integrity was determined using an Agilent Bioanalyzer, with a Bioanalyzer RIN score greater than 8.0 obtained for all samples. ds-cDNA was prepared using the SMARTer Ultra Low RNA kit for Illumina Sequencing (Takara-Clontech) per the manufacturer’s protocol. cDNA was fragmented using a Covaris E220 sonicator using peak incident power 18, duty factor 20%, cycles/burst 50, time 120 seconds to yield an average size of 200 base pairs (bp). cDNA was then blunt ended, had an A base added to the 3’ ends, and then had Illumina sequencing adapters ligated to the ends. Ligated fragments were then amplified for 12 cycles using primers incorporating unique index tags. Fragments were multiplexed with 5-6 samples per lane and were sequenced on an Illumina HiSeq 2500 using single end 50 bp reads to target 30M per sample.

### RNA-Seq Data Acquisition, Quality Control, and Processing

RNA-seq reads from the twenty individual libraries (5 mice per exposure group) were demultiplexed using a custom demultiplexing script written in Python and then aligned to the Ensembl GRCm38.76 (*Mus musculus*) assembly with STAR version 2.0.4b. Gene counts were derived from the number of uniquely aligned unambiguous reads by Subread:featureCount version 1.4.5. Transcript counts were produced by Sailfish version 0.6.3. Sequencing performance was assessed for total number of aligned reads, total number of uniquely aligned reads, genes and transcripts detected, ribosomal fraction, known junction saturation and read distribution over known gene models with RSeQC version 2.3. All gene-level and transcript counts were then imported into the R/Bioconductor package EdgeR and TMM-normalized to adjust for differences in library size. Genes or transcripts not expressed in any sample were excluded from further analysis. Performance of the samples was assessed with a Spearman correlation matrix and multi-dimensional scaling plots. Generalized linear models with robust dispersion estimates were created to test for gene/transcript level differential expression. The fits of the trended and tagwise dispersion estimates were then plotted to confirm proper fit of the observed mean to variance relationship where the tagwise dispersions are equivalent to the biological coefficients of variation of each gene. Differentially expressed genes and transcripts (comparing PBS-1 *vs. Gard*-1 and PBS-2 *vs. Gard*-2) were then filtered for FDR adjusted P values less than or equal to 0.05. For each EdgeR contrast, global perturbations in known Gene Ontology (GO) terms and KEGG pathways were detected using the R/Bioconductor package GAGE to test for changes in expression of the reported log_2_ fold-changes reported by edgeR in each term *versus* the background log_2_ fold-changes of all genes found outside the respective term. The R/Bioconductor package heatmap3 was used to display heatmaps across groups of samples for each GO term with a Benjamini-Hochberg false-discovery rate adjusted P value less than or equal to 0.05.

### Recurrent UTI Experiments in Wild Type and *Nur77* -/- Mice

Mice were anesthetized with isoflurane and then inoculated transurethrally with 50 μL prepared UPEC inoculum. Urine was collected at 24 hpi, and weekly thereafter for 4 weeks. A subset of mice were sacrificed at 2 weeks to compare bladder reservoir titers. Mice that no longer had detectable UTI89 in urine at 4 weeks, reflecting resolution of the initial bladder lumen infection, were used for recurrent UTI experiments. The groups were frequency matched based upon the time course of clearance of UPEC urine titers during the initial infection (O’Brien et al., 2020). Mice were given two bladder exposures of PBS or *Gardnerella*, 1 week apart (transurethral inoculations prepared as in experiments described above). Urine was collected at 24, 48 and 72 h after the second exposure and titers were enumerated by serial dilution and plating on selective media (LB+kanamycin to detect UPEC; NYCIII+streptomycin to detect *Gardnerella*). At 72 h, mice were humanely sacrificed *via* cervical dislocation under isofluorane anaesthesia and bladders and kidneys were aseptically harvested. Homogenates were prepared in 1 mL sterile PBS and plated on appropriate selective media. Bacterial burden in each sample was calculated as CFU/bladder. Samples with no colonies were plotted at one-half of the limit of detection.

## Data Availability Statement

The data discussed in this publication have been deposited in NCBI's Gene Expression Omnibus (Edgar et al., 2002) and are accessible through GEO Series accession number GSE186800 (https://www.ncbi.nlm.nih.gov/geo/query/acc.cgi?acc=GSE186800).

## Ethics Statement

The animal study was reviewed and approved by The Institutional Animal Care and Use Committee (IACUC) of Washington University School of Medicine

## Author Contributions

NG and VO’B performed experiments. NG, VO’B, and AL analyzed the data. NG and VO’B drafted the manuscript. All authors provided funding to support the research. All authors contributed to the article and approved the submitted version.

## Funding

This work was supported by the National Institutes of Health NIAID [R01 AI114635 to AL and R21 AI152049 to AL and NG] and NIDDK [R21 DK092586 to AL and K01 DK110225 to NG], by the National Science Foundation [Graduate Research Fellowship to VO’B #DGE–1143954], by the American Heart Association [Postdoctoral Fellowship to NMG] and by the Center for Women’s Infectious Disease Research at Washington University School of Medicine in St. Louis [Pilot Research Grant to NG]. Some of the animal studies were performed in a facility supported by the NCRR [C06 RR015502]. The funders had no role in study design, data collection and analysis, decision to publish, or preparation of the manuscript.

## Conflict of Interest

The authors declare that the research was conducted in the absence of any commercial or financial relationships that could be construed as a potential conflict of interest.

## Publisher’s Note

All claims expressed in this article are solely those of the authors and do not necessarily represent those of their affiliated organizations, or those of the publisher, the editors and the reviewers. Any product that may be evaluated in this article, or claim that may be made by its manufacturer, is not guaranteed or endorsed by the publisher.
