# Supplementary material for: Bladder Exposure to Gardnerella Activates Host Pathways Necessary for Escherichia coli Recurrent UTI
Source: Front Cell Infect Microbiol. 2021 Dec 6;11:788229. doi: 10.3389/fcimb.2021.788229 (PMC8685330; doi:10.3389/fcimb.2021.788229)
Supplement: Supplementary file 1 [file DataSheet_1.zip › Supplementary Table 1.DOCX]

| Exposure Group | **PBS-1** | | | | | ***Gard*-1** | | | | |
| --- | --- | --- | --- | --- | --- | --- | --- | --- | --- | --- |
| Mouse number | 24 | 42 | 47 | 48 | 49 | 21 | 22 | 23 | 34 | 55 |
| Mapping speed, Million of reads per hour \| | 33.63 | 67.41 | 50.18 | 32.32 | 42.06 | 50.8 | 58.88 | 38.53 | 66.27 | 67.81 |
| Number of input reads \| | 32005047 | 57731402 | 25147397 | 21808138 | 43762980 | 29071087 | 37142530 | 17051535 | 57990103 | 53137583 |
| Average input read length \| | 49 | 49 | 49 | 49 | 49 | 49 | 49 | 49 | 49 | 49 |
| UNIQUE READS: |  |  |  |  |  |  |  |  |  |  |
| Uniquely mapped reads number \| | 21416187 | 42665019 | 15984212 | 13926632 | 29933213 | 21695353 | 27732893 | 12170081 | 42134955 | 40432002 |
| Uniquely mapped reads % \| | 66.92% | 73.90% | 63.56% | 63.86% | 68.40% | 74.63% | 74.67% | 71.37% | 72.66% | 76.09% |
| Average mapped length \| | 48.8 | 49.22 | 48.83 | 48.92 | 48.86 | 49.13 | 49.24 | 49.02 | 49.28 | 49.33 |
| Number of splices: Total \| | 2712235 | 6432895 | 2244240 | 2291228 | 3646089 | 2288476 | 3680886 | 1361778 | 6884568 | 5737700 |
| Number of splices: Annotated (sjdb) \| | 2657704 | 6373019 | 2207284 | 2262548 | 3578473 | 2254103 | 3635664 | 1338693 | 6819429 | 5680166 |
| Number of splices: GT/AG \| | 2692205 | 6386045 | 2230482 | 2277531 | 3619460 | 2270817 | 3653209 | 1351408 | 6838955 | 5696964 |
| Number of splices: GC/AG \| | 14949 | 34110 | 10688 | 9605 | 19911 | 13201 | 20663 | 7823 | 33377 | 29892 |
| Number of splices: AT/AC \| | 1997 | 4617 | 1557 | 1608 | 2676 | 1858 | 3061 | 1017 | 5418 | 4434 |
| Number of splices: Non-canonical \| | 3084 | 8123 | 1513 | 2484 | 4042 | 2600 | 3953 | 1530 | 6818 | 6410 |
| Mismatch rate per base, % \| | 0.56% | 0.20% | 0.42% | 0.32% | 0.61% | 0.34% | 0.27% | 0.41% | 0.25% | 0.22% |
| Deletion rate per base \| | 0.01% | 0.00% | 0.01% | 0.00% | 0.01% | 0.01% | 0.01% | 0.01% | 0.01% | 0.01% |
| Deletion average length \| | 1.45 | 1.5 | 1.5 | 1.67 | 1.51 | 1.41 | 1.47 | 1.42 | 1.53 | 1.44 |
| Insertion rate per base \| | 0.00% | 0.00% | 0.00% | 0.00% | 0.00% | 0.00% | 0.00% | 0.00% | 0.00% | 0.00% |
| Insertion average length \| | 1.14 | 1.13 | 1.14 | 1.16 | 1.12 | 1.12 | 1.11 | 1.13 | 1.13 | 1.12 |
| MULTI-MAPPING READS: |  |  |  |  |  |  |  |  |  |  |
| Number of reads mapped to multiple loci \| | 7215706 | 13249300 | 6830370 | 6834034 | 9525298 | 5749974 | 7785600 | 3529763 | 14003784 | 10896933 |
| % of reads mapped to multiple loci \| | 22.55% | 22.95% | 27.16% | 31.34% | 21.77% | 19.78% | 20.96% | 20.70% | 24.15% | 20.51% |
| Number of reads mapped to too many loci \| | 397298 | 488684 | 345876 | 269075 | 595738 | 377299 | 459084 | 195417 | 496652 | 532745 |
| % of reads mapped to too many loci \| | 1.24% | 0.85% | 1.38% | 1.23% | 1.36% | 1.30% | 1.24% | 1.15% | 0.86% | 1.00% |
| UNMAPPED READS: |  |  |  |  |  |  |  |  |  |  |
| % of reads unmapped: too many mismatches \| | 0.03% | 0.01% | 0.02% | 0.02% | 0.03% | 0.01% | 0.01% | 0.02% | 0.01% | 0.01% |
| % of reads unmapped: too short \| | 8.74% | 1.94% | 7.32% | 3.24% | 7.79% | 3.42% | 2.42% | 6.13% | 2.06% | 1.80% |
| % of reads unmapped: other \| | 0.53% | 0.36% | 0.56% | 0.32% | 0.66% | 0.86% | 0.70% | 0.63% | 0.27% | 0.60% |

| Exposure Group | **PBS-2** | | | | | ***Gard-*2** | | | | |
| --- | --- | --- | --- | --- | --- | --- | --- | --- | --- | --- |
| Mouse number | 20 | 32 | 33 | 56 | 59 | 26 | 27 | 28 | 39 | 60 |
| Mapping speed, Million of reads per hour \| | 44.96 | 42.32 | 72.32 | 53.21 | 73.98 | 37.89 | 37.78 | 56.11 | 33.59 | 78.31 |
| Number of input reads \| | 38716312 | 16633759 | 19947604 | 32031541 | 17324149 | 16428961 | 29751057 | 42722168 | 26660967 | 25559720 |
| Average input read length \| | 49 | 49 | 49 | 49 | 49 | 49 | 49 | 49 | 49 | 49 |
| UNIQUE READS: |  |  |  |  |  |  |  |  |  |  |
| Uniquely mapped reads number \| | 28258675 | 10514108 | 13515740 | 22926463 | 12448186 | 12401376 | 23051087 | 29683262 | 17578671 | 18698846 |
| Uniquely mapped reads % \| | 72.99% | 63.21% | 67.76% | 71.57% | 71.85% | 75.48% | 77.48% | 69.48% | 65.93% | 73.16% |
| Average mapped length \| | 48.97 | 49.4 | 49.19 | 49.22 | 49.3 | 49.32 | 49.27 | 49.01 | 49.03 | 49.11 |
| Number of splices: Total \| | 1981828 | 2103739 | 1989792 | 3412784 | 2084232 | 1918029 | 2563189 | 4373184 | 2762773 | 2496457 |
| Number of splices: Annotated (sjdb) \| | 1935826 | 2082965 | 1971029 | 3372041 | 2064677 | 1897197 | 2531403 | 4315016 | 2725763 | 2464923 |
| Number of splices: GT/AG \| | 1967031 | 2090572 | 1978564 | 3388833 | 2070337 | 1903167 | 2544039 | 4344508 | 2744678 | 2478988 |
| Number of splices: GC/AG \| | 10656 | 9762 | 7861 | 17590 | 10050 | 11259 | 14384 | 21322 | 13723 | 12908 |
| Number of splices: AT/AC \| | 1536 | 1551 | 1308 | 2665 | 1612 | 1464 | 2014 | 3221 | 2156 | 1888 |
| Number of splices: Non-canonical \| | 2605 | 1854 | 2059 | 3696 | 2233 | 2139 | 2752 | 4133 | 2216 | 2673 |
| Mismatch rate per base, % \| | 0.61% | 0.22% | 0.23% | 0.25% | 0.26% | 0.21% | 0.37% | 0.45% | 0.37% | 0.35% |
| Deletion rate per base \| | 0.01% | 0.00% | 0.01% | 0.01% | 0.01% | 0.01% | 0.01% | 0.01% | 0.01% | 0.01% |
| Deletion average length \| | 1.35 | 1.58 | 1.64 | 1.54 | 1.44 | 1.49 | 1.36 | 1.47 | 1.49 | 1.41 |
| Insertion rate per base \| | 0.00% | 0.00% | 0.00% | 0.00% | 0.00% | 0.00% | 0.00% | 0.00% | 0.00% | 0.00% |
| Insertion average length \| | 1.1 | 1.13 | 1.12 | 1.12 | 1.13 | 1.11 | 1.11 | 1.14 | 1.14 | 1.12 |
| MULTI-MAPPING READS: |  |  |  |  |  |  |  |  |  |  |
| Number of reads mapped to multiple loci \| | 6894618 | 4496294 | 5401320 | 7857927 | 4239381 | 3532162 | 5366473 | 10390872 | 7545486 | 5435264 |
| % of reads mapped to multiple loci \| | 17.81% | 27.03% | 27.08% | 24.53% | 24.47% | 21.50% | 18.04% | 24.32% | 28.30% | 21.26% |
| Number of reads mapped to too many loci \| | 872498 | 148172 | 263813 | 347815 | 176636 | 136576 | 362878 | 473918 | 316287 | 295118 |
| % of reads mapped to too many loci \| | 2.25% | 0.89% | 1.32% | 1.09% | 1.02% | 0.83% | 1.22% | 1.11% | 1.19% | 1.15% |
| UNMAPPED READS: |  |  |  |  |  |  |  |  |  |  |
| % of reads unmapped: too many mismatches \| | 0.03% | 0.00% | 0.01% | 0.01% | 0.00% | 0.01% | 0.01% | 0.02% | 0.01% | 0.01% |
| % of reads unmapped: too short \| | 5.34% | 8.58% | 3.10% | 2.31% | 2.28% | 1.86% | 2.42% | 4.73% | 4.22% | 3.86% |
| % of reads unmapped: other \| | 1.58% | 0.28% | 0.74% | 0.49% | 0.37% | 0.32% | 0.83% | 0.35% | 0.34% | 0.55% |
