# Supplementary material for: Bladder Exposure to Gardnerella Activates Host Pathways Necessary for Escherichia coli Recurrent UTI
Source: Front Cell Infect Microbiol. 2021 Dec 6;11:788229. doi: 10.3389/fcimb.2021.788229 (PMC8685330; doi:10.3389/fcimb.2021.788229)
Supplement: Supplementary file 1 [file DataSheet_1.zip › Supplementary Tables 6-8.PDF]

Supplementary Table 6. KEGG Pathways Differentially Expressed in *Gard*-1 vs. PBS-1	log2 fold change	P-value
**Immune System**		
mmu04060 Cytokine-cytokine receptor interaction	-3.9074244	5.39E-05
mmu04062 Chemokine signaling pathway	-3.855439	7.19E-05
mmu04612 Antigen processing and presentation	-3.7392598	0.000136382
mmu04672 Intestinal immune network for IgA production	-2.8544873	0.002817565
mmu04660 T cell receptor signaling pathway	-2.7784717	0.003103109
mmu04650 Natural killer cell mediated cytotoxicity	-2.6918667	0.003868196
mmu04640 Hematopoietic cell lineage	-2.206054	0.014481619
mmu04662 B cell receptor signaling pathway	-2.0402452	0.022233554
**Metabolism**		
mmu00830 Retinol metabolism	2.64860079	0.004417632
mmu00591 Linoleic acid metabolism	2.43147347	0.008552663
mmu00140 Steroid hormone biosynthesis	1.99609712	0.023814008
mmu00590 Arachidonic acid metabolism	1.88376244	0.03070571
mmu00190 Oxidative phosphorylation	-4.7134587	2.03E-06
mmu00480 Glutathione metabolism	-2.3856795	0.01000639
mmu00240 Pyrimidine metabolism	-2.3286051	0.01072116
mmu00010 Glycolysis / Gluconeogenesis	-2.0372119	0.022217838
mmu00620 Pyruvate metabolism	-1.8421649	0.03572698
mmu00230 Purine metabolism	-1.815900	0.035368769
**Genetic Information Processing**		
mmu03010 Ribosome	-8.4850097	3.91E-15
mmu03050 Proteasome	-3.0582704	0.001604383
mmu03040 Spliceosome	-2.5962792	0.005142505

mmu03060 Protein export	-2.0064456	0.025893787
mmu03013 RNA transport	-1.9331562	0.027217075
mmu03030 DNA replication	-1.7957583	0.040141152
mmu03420 Nucleotide excision repair	-1.7749877	0.041163226
mmu03020 RNA polymerase	-1.757043	0.043690654
**Signalling Molecules and Interaction**		
mmu04080 Neuroactive ligand-receptor interaction	1.88795095	0.029802399
mmu04514 Cell adhesion molecules (CAMs)	-2.5036058	0.00642753
**Signal Transduction**		
mmu04064 NF-kappa B signaling pathway	-2.1812431	0.015459215
mmu04664 Fc epsilon RI signaling pathway	-1.8529596	0.033494299
**Cellular Processes**		
mmu04142 Lysosome	-1.8321632	0.034353597
mmu04146 Peroxisome	-1.7504744	0.041467489
mmu04145 Phagosome	-2.8299623	0.002482533

**Supplementary Table 7. KEGG Pathways Differentially Expressed in *Gard*-2 vs. PBS-2**	**log2 fold change**	**P-value**
**Urothelial Integrity**		
mmu04510 Focal adhesion	6.25568449	6.93E-10
mmu04810 Regulation of actin cytoskeleton	4.68984271	1.95E-06
mmu04520 Adherens junction	4.88343863	2.46E-06
mmu04512 ECM-receptor interaction	3.30225354	0.00060658
mmu04530 Tight junction	3.25090109	0.00066448
mmu04514 Cell adhesion molecules (CAMs)	3.13967931	0.00094215
mmu04540 Gap junction	2.63688994	0.00470922
mmu00532 Glycosaminoglycan biosynthesis - chondroitin sulfate / dermatan sulfate	1.93823931	0.03346704
mmu00534 Glycosaminoglycan biosynthesis - heparan sulfate / heparin	1.85774807	0.03711655
**Immune System**		
mmu04668 TNF signaling pathway	4.03223777	4.84E-05
mmu04666 Fc gamma R-mediated phagocytosis	3.90237412	9.18E-05
mmu04350 TGF-beta signaling pathway	3.50038207	0.00033435
mmu04660 T cell receptor signaling pathway	3.45525886	0.00037486
mmu04650 Natural killer cell mediated cytotoxicity	3.42889535	0.00039028
mmu04670 Leukocyte transendothelial migration	3.25800056	0.00066155
mmu04620 Toll-like receptor signaling pathway	3.22993672	0.0007913
mmu04622 RIG-I-like receptor signaling pathway	3.19847999	0.00094944
mmu04662 B cell receptor signaling pathway	3.05967056	0.00151523
mmu04062 Chemokine signaling pathway	3.03738643	0.00132135
mmu04066 HIF-1 signaling pathway	2.93242434	0.0019341
mmu04060 Cytokine-cytokine receptor interaction	2.89781515	0.00198847

mmu04664 Fc epsilon RI signaling pathway	2.57988293	0.00577153
mmu04612 Antigen processing and presentation	2.29458387	0.0118012
mmu04621 NOD-like receptor signaling pathway	1.98518845	0.02567241
mmu04640 Hematopoietic cell lineage	1.9394848	0.02732158
**Signaling Pathway**		
mmu04151 PI3K-Akt signaling pathway	5.0988416	2.34E-07
mmu04722 Neurotrophin signaling pathway	4.83357159	1.74E-06
mmu04910 Insulin signaling pathway	4.61032589	3.45E-06
mmu04070 Phosphatidylinositol signaling system	4.4558195	1.16E-05
mmu04012 ErbB signaling pathway	4.21166057	2.92E-05
mmu04915 Estrogen signaling pathway	3.98667001	5.56E-05
mmu04010 MAPK signaling pathway	3.86890771	6.38E-05
mmu04630 Jak-STAT signaling pathway	3.89732942	6.40E-05
mmu04912 GnRH signaling pathway	3.72093389	0.00015192
mmu04150 mTOR signaling pathway	3.79538697	0.00015223
mmu04390 Hippo signaling pathway	3.55270328	0.000225
mmu04064 NF-kappa B signaling pathway	3.32677028	0.00060423
mmu04330 Notch signaling pathway	2.91188045	0.00232546
mmu04115 p53 signaling pathway	2.79865099	0.00332013
mmu04310 Wnt signaling pathway	2.6357592	0.00446609
mmu04370 VEGF signaling pathway	2.34557949	0.01079771
mmu04020 Calcium signaling pathway	2.19302675	0.01452552
mmu04917 Prolactin signaling pathway	2.11422046	0.01839216
mmu04723 Retrograde endocannabinoid signaling	2.09957059	0.01866483
**Metabolism**		
mmu00564 Glycerophospholipid metabolism	3.68165233	0.00018015

mmu00562 Inositol phosphate metabolism	3.27897631	0.00076756
mmu00280 Valine, leucine and isoleucine degradation	2.77609834	0.00355034
mmu00310 Lysine degradation	2.73458108	0.0040218
mmu00970 Aminoacyl-tRNA biosynthesis	2.59410537	0.00640848
mmu00240 Pyrimidine metabolism	2.40175835	0.0088945
mmu00563 Glycosylphosphatidylinositol(GPI)-anchor biosynthesis	2.21306811	0.01801211
mmu00230 Purine metabolism	2.19253602	0.01460151
mmu00561 Glycerolipid metabolism	1.94613251	0.02732353
mmu00510 N-Glycan biosynthesis	1.92392778	0.02938892
mmu00450 Selenocompound metabolism	1.96038873	0.03310137
mmu00830 Retinol metabolism	-2.5103455	0.00649925
mmu00140 Steroid hormone biosynthesis	-1.9318109	0.02762109
**Genetic Information Processing**		
mmu04120 Ubiquitin mediated proteolysis	5.33572742	1.68E-07
mmu04141 Protein processing in endoplasmic reticulum	4.6692388	2.53E-06
mmu03040 Spliceosome	3.90456613	6.97E-05
mmu03013 RNA transport	3.40860055	0.00037873
mmu03015 mRNA surveillance pathway	2.91003464	0.00208583
mmu03018 RNA degradation	2.81190429	0.0029107
mmu03420 Nucleotide excision repair	2.18300185	0.01725776
mmu03410 Base excision repair	2.01414902	0.02585006
mmu03440 Homologous recombination	1.97876877	0.0289198
mmu03460 Fanconi anemia pathway	2.75789899	0.00391604
mmu03030 DNA replication	1.85262033	0.03600018
**Cellular Processes**		
mmu04144 Endocytosis	6.13666639	1.25E-09

mmu04110 Cell cycle	4.1265121	3.06E-05
mmu04114 Oocyte meiosis	3.92220392	6.71E-05
mmu04210 Apoptosis	3.21631964	0.00089053
mmu04142 Lysosome	3.2122798	0.00079821
mmu04146 Peroxisome	2.76901811	0.00326449
mmu04130 SNARE interactions in vesicular transport	1.8202926	0.03911179
**Other Organismal Systems**		
mmu04360 Axon guidance	4.25980659	1.64E-05
mmu04728 Dopaminergic synapse	4.04843796	3.72E-05
mmu04380 Osteoclast differentiation	3.5497723	0.00025121
mmu04725 Cholinergic synapse	3.37048131	0.00047393
mmu04914 Progesterone-mediated oocyte maturation	3.39057401	0.00048953
mmu04270 Vascular smooth muscle contraction	3.03161861	0.00136232
mmu04710 Circadian rhythm	3.04024319	0.00210459
mmu04720 Long-term potentiation	2.8889109	0.00238319
mmu04971 Gastric acid secretion	2.75696433	0.00337774
mmu04724 Glutamatergic synapse	2.72855052	0.00352184
mmu04970 Salivary secretion	2.72834938	0.00363567
mmu04962 Vasopressin-regulated water reabsorption	2.51820281	0.00724934
mmu04713 Circadian entrainment	2.08620884	0.01934447
mmu04961 Endocrine and other factor-regulated calcium reabsorption	1.99792873	0.02429088
mmu04320 Dorso-ventral axis formation	1.78780581	0.04089672
**Other**		
mmu04730 Long-term depression	2.37137938	0.00988271
mmu02010 ABC transporters	1.98937165	0.02528137
mmu04960 Aldosterone-regulated sodium reabsorption	1.98909226	0.0257273

mmu04140 Regulation of autophagy	1.95171057	0.02795451
mmu04721 Synaptic vesicle cycle	1.93169559	0.02829394

Supplementary Table 8. Genes Increased 12 h After *Gardnerella* Exposure

**Gene**	**Description**	**logCPM PBS-1** (mean +/- SEM)	**logCPM *Gard-1*** (mean +/- SEM)	**log2FC**	**P Value**	**FDR**
Nr4a1 aka Nur77	nuclear receptor subfamily 4, group A, member 1	3.465 +/- 0.13	5.928 +/- 0.44	2.5138	1.47E-10	5.25E-06
Atf3	activating transcription factor 3	2.526 +/- 0.37	5.206 +/- 0.49	2.8361	1.49E-08	2.66E-04
Arc	activity regulated cytoskeletal-associated protein	-0.3284 +/- 0.25	1.640 +/- 0.54	2.2063	1.80E-06	2.15E-02
Nr4a2 aka Nurr1	nuclear receptor subfamily 4, group A, member 2	2.025 +/- 0.19	3.585 +/- 0.43	1.6333	3.25E-06	2.90E-02
Fosb	FBJ osteosarcoma oncogene B	1.752 +/- 0.27	4.296 +/- 0.69	2.6821	5.13E-06	3.67E-02
